# Lithophagia in a 4-Year-Old Child

**DOI:** 10.7759/cureus.83334

**Published:** 2025-05-02

**Authors:** Meriem El achiwi, Fatimazahra Yakine, Fatima zahra Alaoui-Inboui, Salimi Soundouss, Bouchra Slaoui

**Affiliations:** 1 Pediatrics Department 2, Pulmonology and Allergology Unit, Hôpital Mère-Enfant Abderrahim Harouchi, Casablanca, MAR; 2 Pediatrics Department 2, Pulmonology and Allergology Unit, Hôpital Mère-Enfants Abderrahim Harouchi, Casablanca, MAR; 3 Pediatrics Department 2, Pulmonology and Allergology Unit, Hôpital Mère-Enfants Abderrahim Harouchi, casablanca, MAR

**Keywords:** eating behavior disorder, foreign body ingestion, lithophagia, pediatrics, pica

## Abstract

Lithophagia is a form of pica syndrome, characterized by the ingestion of stones. It is an eating behavior disorder primarily observed in children and often associated with neurodevelopmental disorders or nutritional deficiencies. This condition may lead to severe complications such as intestinal obstruction, gastrointestinal perforation, or even acute bowel obstruction requiring prompt and appropriate medical management.

We report the case of a 4-year-old girl who was initially hospitalized for hydrocarbon poisoning. Upon admission, the patient presented with a fever of 39°C, acute respiratory distress with severe tachypnea at 45 breaths per minute, and signs of respiratory struggle. Chest radiography revealed an alveolar consolidation in the left lower lobe, suggestive of aspiration pneumonia.

In addition, the patient complained of severe abdominal pain, prompting an abdominal ultrasound, which returned without abnormalities. The clinical course was marked by worsening of the abdominal symptoms, with cessation of stool and gas passage, leading to the performance of a plain abdominal X-ray. The imaging revealed multiple radio-opaque objects scattered throughout the intestinal tract, highly suggestive of foreign body ingestion.

Symptomatic treatment was initiated, including close clinical monitoring and stool surveillance, which led to the passage of stones in the feces, thereby confirming the diagnosis of lithophagia. Furthermore, the child showed no signs of anemia, and her serum ferritin levels were within the normal range. Zinc level testing was indicated.

## Introduction

Lithophagia, a variant of pica syndrome, is an eating behavior disorder characterized by the ingestion of stones [1]. This condition can lead to serious complications such as intestinal obstruction or gastrointestinal perforation [2,3]. Diagnosis is based on clinical history and imaging studies, particularly plain abdominal radiography [4]. Management generally includes nutritional education and, if necessary, laxatives or medical procedures to extract ingested foreign bodies [3,5]. Early intervention is essential to prevent complications.

We report the case of a 4-year-old girl admitted for hydrocarbon poisoning who developed acute abdominal pain related to lithophagia.

## Case presentation

A 4-year-old girl was admitted for hydrocarbon poisoning. On examination, she was febrile at 39°C, tachypneic at 45 breaths per minute, and tachycardic at 110 beats per minute, with signs of respiratory distress but no adventitious sounds on lung auscultation.

Chest radiography revealed an alveolar infiltrate in the left lower lobe. The patient was stabilized and started on antibiotic therapy with amoxicillin-clavulanic acid.

**Figure 1:**
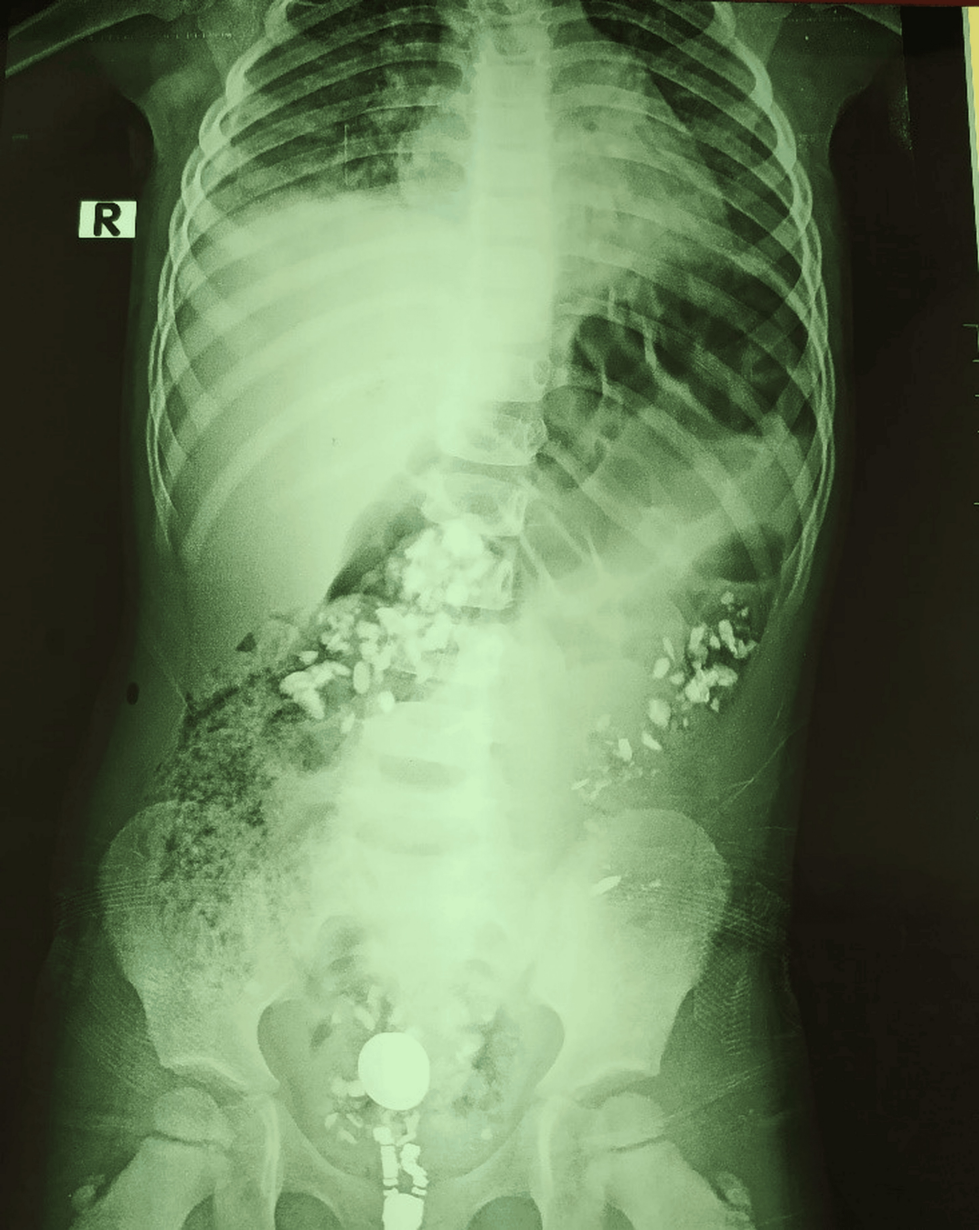
Plain abdominal X-ray showing multiple radio-opaque densities along the colon.

In parallel, the patient also complained of diffuse abdominal pain, prompting an abdominal ultrasound, which revealed no abnormalities. Clinical progression was marked by worsening abdominal pain, associated with cessation of stool and gas passage. On examination, the abdomen was soft and non-distended but diffusely tender. In light of this presentation, a plain abdominal X-ray was performed, revealing multiple radiopaque images along the colon, as can be seen in Figure 1.

**Figure 2:**
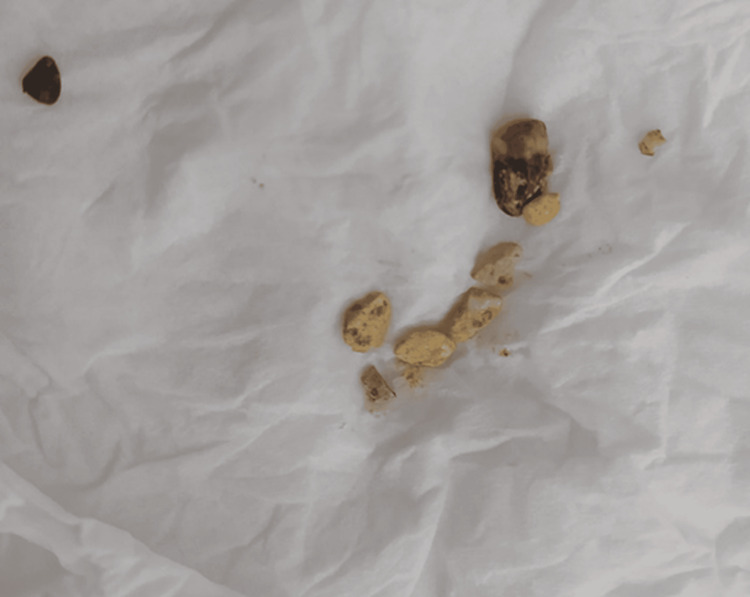
Progressive evacuation of ingested stones following laxative therapy.

A surgical consultation was requested due to suspected stone ingestion. Upon further questioning, the mother reported a history of geophagia. The patient was treated with laxatives and a micro-enema. Following the passage of the first stools, several small stones were observed, and further cleansing led to the evacuation of numerous pebbles, as illustrated in the image below in Figure 2.

The complete blood count did not reveal any anemia, and serum ferritin was 79 ng/mL. Zinc levels were not assessed.

## Discussion

Pica syndrome is defined by the American Psychiatric Association as the persistent ingestion of non-food, non-nutritive substances for a duration of at least one month in a child aged 24 months or older [6]. This phenomenon is primarily observed in children, often in the context of nutritional deficiencies, developmental disorders, or psychiatric conditions. Pica has been associated with iron and zinc deficiency, as well as cognitive disorders such as autism spectrum disorder and intellectual disability [6,7]. It is essential to distinguish pica from normal exploratory behavior in young children, such as mouthing objects, in order to establish an accurate diagnosis and prevent serious complications.

Lithophagia, or the ingestion of stones, is a specific form of pica commonly seen in children, particularly those with autism spectrum disorders. Several complications may arise, including colitis, intestinal obstruction, and volvulus, which often require surgical intervention [3].

In the context of pica, a comprehensive evaluation is necessary to exclude other underlying causes. For instance, children with nutritional deficiencies should undergo a full nutritional assessment to correct these imbalances, particularly in iron and zinc levels [8,9]. Additionally, investigations should be conducted to explore psychological or developmental causes of disordered eating behavior, especially in cases where autism spectrum disorder or intellectual disability is suspected [6,10].

This patient presented with lithophagia in the absence of any psychiatric history or iron deficiency, making it an atypical case rarely described in the literature. Zinc level assessment is warranted.

Narayanan et al. reported the case of a 10-year-old child with behavioral disturbances who was admitted for acute abdominal pain mimicking colitis. The etiology was found to be chronic ingestion of foreign bodies (gravel and stones) accumulated in the colon over a year. Mechanical rectal extraction failed, and surgery was considered; however, resolution was achieved through iterative colonoscopic evacuations, antibiotic therapy, and laxatives [11]. In our case, the outcome was favorable with conservative treatment and no surgical complications.

Another case was described by Cross and Holland, involving a 16-month-old infant with no relevant medical history, admitted for non-bilious vomiting, fever, somnolence, and irritability. Imaging suggested appendicitis, but laparotomy revealed an ileal perforation with a subserosal abscess containing small metallic foreign bodies. Segmental ileal resection and appendectomy were performed [2].

The management of pica and lithophagia requires a multidisciplinary approach. Treatment may include symptom-oriented measures such as laxatives for gastrointestinal complications and referral to behavioral therapies, such as cognitive behavioral therapy [10,12]. In cases of deficiency, iron and zinc supplementation is generally recommended [9].

The case presented highlights the importance of early diagnosis and intervention to prevent serious complications associated with lithophagia. Prompt management, combined with nutritional and behavioral evaluation, can significantly improve the prognosis [3].

## Conclusions

Lithophagia is a rare and often underdiagnosed form of pica, posing significant diagnostic challenges in pediatric practice. Early recognition is essential to prevent potentially serious complications such as gastrointestinal obstruction, perforation, or infection. Beyond behavioral disorders, nutritional deficiencies - particularly in iron and zinc - may contribute to the onset of lithophagia. Although management is generally conservative, surgical intervention may become necessary in cases of complications. A comprehensive approach combining early diagnosis, nutritional assessment, and close clinical monitoring is crucial for improving outcomes in affected children.
